# Lipid Nanoparticulate Drug Delivery Systems: Recent Advances in the Treatment of Skin Disorders

**DOI:** 10.3390/ph14111083

**Published:** 2021-10-26

**Authors:** Stefan R. Stefanov, Velichka Y. Andonova

**Affiliations:** Department of Pharmaceutical Technologies, Faculty of Pharmacy, Medical University of Varna, 9002 Varna, Bulgaria; Velichka.Andonova@mu-varna.bg

**Keywords:** skin diseases, lipid-based nanosystems, solid lipid nanoparticles, nanostructured lipid carriers, cream, ointment, gel

## Abstract

The multifunctional role of the human skin is well known. It acts as a sensory and immune organ that protects the human body from harmful environmental impacts such as chemical, mechanical, and physical threats, reduces UV radiation effects, prevents moisture loss, and helps thermoregulation. In this regard, skin disorders related to skin integrity require adequate treatment. Lipid nanoparticles (LN) are recognized as promising drug delivery systems (DDS) in treating skin disorders. Solid lipid nanoparticles (SLN) together with nanostructured lipid carriers (NLC) exhibit excellent tolerability as these are produced from physiological and biodegradable lipids. Moreover, LN applied to the skin can improve stability, drug targeting, occlusion, penetration enhancement, and increased skin hydration compared with other drug nanocarriers. Furthermore, the features of LN can be enhanced by inclusion in suitable bases such as creams, ointments, gels (i.e., hydrogel, emulgel, bigel), lotions, etc. This review focuses on recent developments in lipid nanoparticle systems and their application to treating skin diseases. We point out and consider the reasons for their creation, pay attention to their advantages and disadvantages, list the main production techniques for obtaining them, and examine the place assigned to them in solving the problems caused by skin disorders.

## 1. Introduction

Skin diseases cause significant discomfort to millions of people around the world daily. Various studies show that between 30 and 70% of the world’s population suffers from skin diseases [1]. In most cases, skin diseases are caused mainly by various infectious pathogens (bacteria, fungi, viruses) or inflammatory processes of various etiologies [2,3]. The majority of skin diseases are acute and have a significant psychological impact on individuals. Despite substantial advances in dermatological treatment, many infectious skin problems remain complex and persistent in treatment. These problems depend on the type of pathogen, the integrity of the skin layers, and especially on the patient’s medical status [4]. A number skin diseases such as atopic dermatitis, allergic contact dermatitis, and psoriasis are chronic and represent a complex result of infiltration of inflammatory T cells and increased production of cytokines in the lesions [5]. The success of topical treatment of skin diseases requires a timely, accurate diagnosis, as well as an effective, simple, and not very invasive targeted topical treatment. In addition, it depends on the type of dosage form (the type of delivery system used to supply the drug to the skin) and the application method [6].

This review focuses on the recent advances of lipid-based nanosystems in the treatment of skin disorders. We discuss the most common types of lipid-based nanocarriers researched for dermal drug delivery and used for the treatment of skin disorders by incorporation into appropriate dosage forms, namely: Nanovesicular carriers, lipid nanoparticulate carriers, microemulsions, and nanoemulsions. The considered compositions are intended principally for local treatment and are an attempt to reflect the current picture of development in this field.

## 2. Skin

The skin is the largest metabolically active organ of the human body. Its vital functions include protection from external environmental threats, vitamin D synthesis, and the maintenance of the body’s dynamic balance [7,8,9]. Moreover, the protective function is expressed by limiting the direct invasion of microorganisms as a physical barrier [10]. This defensive mechanism provides physical and immunological, metabolic, and UV protection [11]. Good knowledge of the barrier properties of the skin and the assessment of changes in the barrier functions as a result of skin diseases can be used to successfully develop new effective drug delivery systems, especially for the diseased skin and for the application of therapeutic agents such as drugs or vaccines [12,13]. Topical pharmaceuticals for the treatment of skin disorders can reach the problematic site without the risk of massive systemic absorption or other lateral effects [14].

In short, the skin consists of three main layers—epidermis, dermis, and hypodermis (subcutaneous fat tissue) [15].

The superficial part of the skin is called the epidermis. In essence, it is a laminar, squamous corneal epithelium composed mostly of two types of cells: Keratinocytes and dendritic cells (antigen-presenting cells) [16].

**Figure 1:**
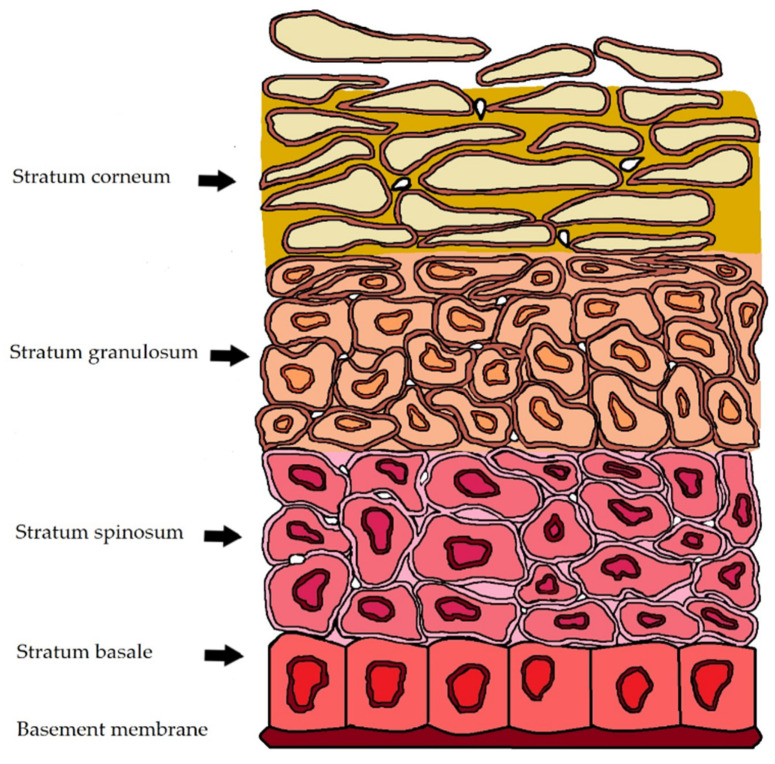
Schematic representation of the epidermis. Epidermis has a thickness of 0.1–0.2 mm. Stratum corneum (10–20 μm) and the viable epidermis (50–150 μm).

The epidermis consists of five layers—the stratum corneum, stratum lucidum, stratum granulosum (granular ply), stratum spinosum (spinous ply), and stratum germinativum (basal ply) [17]. No blood vessels were found in the epidermis [18]. The structure of the epidermis is schematically represented in Figure 1.

**Figure 2:**
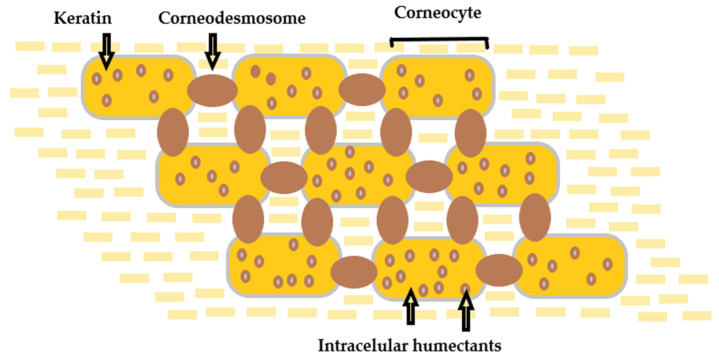
Structure of the *stratum corneum*—“brick and mortar” formation. The pathway between the stacked corneocytes is filled with “mortar” lipids.

The outermost sublayer of the epidermis, named *stratum corneum* (SC) (10–20 µm), plays a fundamental role as the body’s first and main physical skin barrier from external menaces [19,20,21]. It consists of corneocytes—specific cells that are the essential limiting factor for permeation through the skin, restricting the passage of molecules significantly larger than 500 Da [22]. The SC functions as a two-compartment system organized in a “brick and mortar” formation, with an extracellular matrix of lamellar membranes [23]. The structure of the stratum corneum is schematically represented in Figure 2.

The permeation of matter through the SC is possible primarily through passive diffusion in three ways: Transcellular (the bulk of flux), intercellular, and appendageal [24,25,26]. For all of the substances permeating the skin, diffusion through the SC is a rate-limiting stage [27]. In this regard, significant efforts are being made to establish and improve skin nanoparticle delivery systems that can supply and sustainably release active substances of varying lipophilicity and molecular weights to and through the skin, as well as ensure their protection against skin metabolism [28].

Keratinocytes, melanocytes (melanin producers), Merkel cells (sensory receptors), and Langerhans cells (immunocompetent cells) are epidermal formations—essential for the skin’s vitality [29]. In addition, it is crucial to consider the barrier nature of the diseased skin, since there are substantial differences in barrier abilities between healthy and diseased skin. Therefore, we have reason to say that the skin reflects a person’s health.

## 3. Skin Disorders

**Figure 3:**
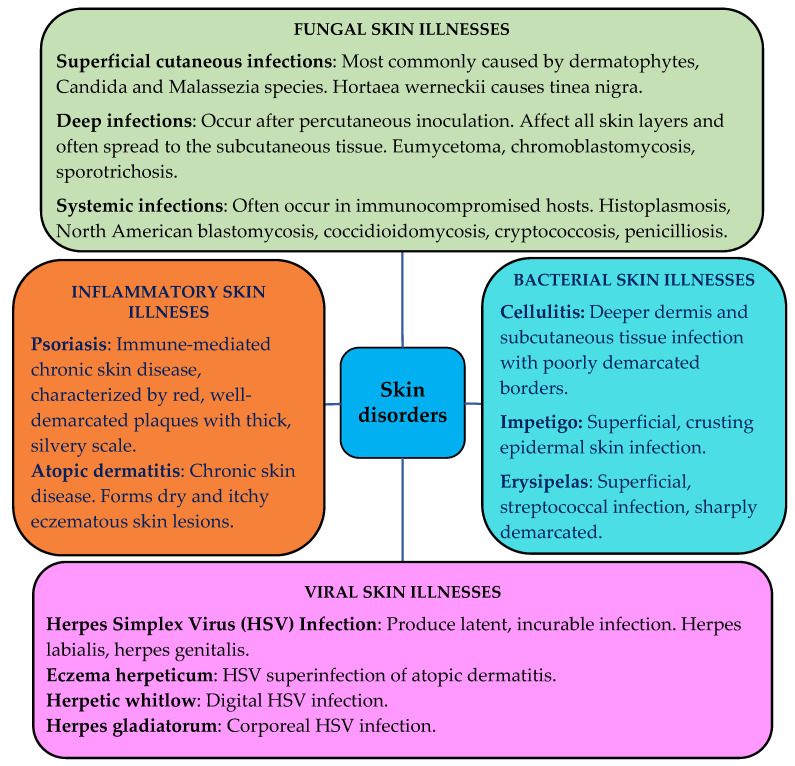
Representation of the most common skin disorders [32,33,34,35,36,37,38,39,40,41,42,43].

The skin can be affected by various pathological changes, i.e., inflammatory, neoplastic, traumatic, hormonal, degenerative, and even hereditarily determined [30]. Infectious skin diseases such as bacterial, fungal or viral affect people and cause various dermatological problems. Chronic inflammatory skin diseases such as atopic dermatitis, allergic contact dermatitis, psoriasis, etc., are a consequence of infiltration of inflammatory T cells [31]. The schematic representation of various skin disorders is shown in Figure 3 [32,33,34,35,36,37,38,39,40,41,42,43].

In practice, most of the skin disorders are complicated, polygenic, and multifactorial [44]. This indicates that multiple factors, lifestyle, and the environment play a fundamental role in the clinical picture of the diseases [45].

### Treatment of Skin Disorders with Conventional Topical Delivery Systems

The human skin creates a vast opportunity for drug delivery application. In general, dermal (topical) and transdermal skin drug delivery can be differentiated. Dermal delivery is the application of the drug directly at the place of action—on the skin’s surface. Transdermal drug delivery is an alternative, painless, and non-invasive approach used to deliver drugs for therapeutic use [46].

Many conventional topical preparations are intended for topical delivery of the drug and not for systemic action. This skin preparation delivers a concentrated amount of the active ingredient for absorption via the application layer [47]. In addition, the use of penetration enhancers in conventional formulations increases the delivery rate through the epidermis, but can also cause unwanted side effects [48].

Some of the commonly used therapeutic solutions are listed in Table 1.

For chronic inflammatory skin diseases, a topical treatment is often not too efficient. Therefore, more efficacious medicinal products (MP) are given systemically, where they can be immunosuppressive, and their long-term usage is not recommended as they suppress the affected area [58].

A large part of therapeutic indications is treated by MP, which are intended for topical administration [59]. These MP feature different molecular structures, but retain some common physicochemical characteristics such as high lipophilicity and poor aqueous solubility. For them, the rate and extent of drug delivery have to be sufficient to achieve local therapeutic concentrations in an acceptable term and provide an effective pharmacological action [60].

The barrier function of the targeted biologic membrane provides a significant challenge for optimal therapy. The efficacy of drug delivery and the therapeutic effect depend mainly on the diffusion affinity of the drug substance and the interaction between the excipients of the formulation and the membrane components. Therefore, the conducive balance between potency and deliverability has to be ensured through the design and development of a delivery system to reach optimum therapeutic levels at the site of infection [60].

Biologics have become increasingly popular as a targeted treatment. Biologics are products composed of sugars, proteins, nucleic acids or complex combinations of these substances. Several biologics that target specific subgroups of cells in the skin have been tested [61].

Empirical experience shows that conventional topical preparations suffer from certain limitations and are compromised in patient compliance, safety, and efficacy of therapy [62]. Against this background, the need for advanced carriers that could effectively improve skin penetration and reduce drug-related side effects is more than necessary [63].

An inventive strategy for improving the penetration of molecules through the epidermal barrier is the application of nanocarriers due to the advantage of their lipophilicity, which mediates the passage through the intact lipid layer. The use of lipid nanoparticles in dermal formulations provides several benefits: Chemical protection of the incorporated drug molecules, application to the skin of labile drug substances, improved bioavailability of drugs, and the ability for better release by provision penetration and retention in the skin [64].

Lipid-based nanosystems have proven to be suitable for dermal carriers due to their biocompatibility, efficient delivery of active ingredients, and stability. In addition, their enhanced surface leads to the improved penetration of active ingredients [65].

## 4. Lipid-Based Drug Delivery Systems

Lipid-based drug delivery systems (LBDDS) are formulations containing a dissolved or suspended drug substance in lipidic excipients [66]. LBDDS are a progressive strategy to formulate pharmaceuticals for topical delivery [67]. Liposomes, which are “pioneers” among lipid DDS, have been used to improve drug solubility and traditionally for topical and transdermal drug delivery.

Table 2 presents a brief overview of lipid-based drug delivery systems.

## 5. Nanovesicular Carriers

Liposomes (further accepted as conventional liposomes) are considered to be the first generation of nanovesicular carriers. They are small artificial vesicles of the spherical shape created from cholesterol and natural, non-toxic phospholipids with an enclosed inner aqueous core [81]. There are several methods for liposome preparation such as thin-film hydration, reverse phase evaporation, and microfluidic mixing [82]. In 1980, Mezei and Gulasekharam reported the first generation of liposomes as drug delivery systems for topical administration [83]. Since then, liposomes are widely applied as dermal drug carriers [84].

The second generation of nanovesicular carriers—transfersomes were developed in 1992 by Cevc et al. They are modified liposomes with an average diameter below 300 nm and contain an edge activator that makes transfersomes nearly eight times more flexible than the conventional liposomes [85,86,87]. Edge activators can be sorbitan esters, sodium cholate, polysorbates, dipotassium glycyrrhizinate, etc. [88]. The competitive advantage of transfersomes is their ability to squeeze through tiny holes, five to ten times smaller than the vesicles dimensions [89,90,91,92]. In 1996, Touitou designed the ethosomes—the next step for improving permeation abilities [93]. These contain a fluid bilayer in their structure due to the high concentration of ethanol—20 to 45 wt%. Ethosomes potentiate the penetration effect through the clogged and unlocked skin and lead to drug penetration to a depth of about 200 µm [94]. Numerous studies have been devoted to the efficacy of ethosomes for dermal drug delivery in both occlusive and non-occlusive applications [95,96]. They demonstrate an excellent ability to improve the skin permeation of drugs, both highly lipophilic and highly hydrophilic active ingredients [97,98,99,100,101]. A certain number of studies are reported for the superior skin delivery of ethosomes compared with liposomes, transferosomes, and commercial formulations [102]. For example, psoralen-loaded ethosomes (an antipsoriasis drug) have shown 3.50 and 2.15 times higher permeation flux and skin deposition, respectively, compared with liposomes [103].

Table 3 summarizes some of the patents for the application of ethosomal drug delivery.

### 5.1. Emerging Lipid Nanovesicular Carriers

The usefulness of different lipid vesicles prompted researchers to experiment with modifications to give them specific structural or application properties [104]. However, the current research on these vesicles is somewhat limited, especially for topical drug delivery. Table 4 lists the emerging lipid vesicles developed for skin drug delivery, in the recent past.

Another nanovesicular carrier widely used for dermal drug delivery is called niosomes, with an average particle size between 50 and 200 nm [106]. These are based on nonionic surfactants and cholesterol and are considered more stable and less expensive than liposomes. Several methods can be used for niosomes preparation such as high-pressure homogenization, extrusion or sonication. Due to the small size of these vesicles, the drug loading and stability decrease. The problem can be solved by adding a stabilizer [121] that can be specified, as well as other nanovesicular carriers that are being increasingly explored in recent years for dermal delivery such as cubosomes, hexasomes, aquasomes, colloidosomes, sphingosomes, ufasomes, archeosomes, lipoplexes, proliposomes, etc. [111,122]. Cubosomes are bicontinuous cubic liquid crystalline phases with two different hydrophilic areas separated by a lipid bilayer [123]. Their preparation requires the usage of high-energy dispersion techniques [124,125]. Hexosomes are built from hexagonal liquid crystalline phases dispersed in a continuous aqueous medium [126]. Cubosomes and hexosomes can incorporate hydrophilic, hydrophobic, and amphiphilic drugs, increased drug loading, and good stability. It has recently been shown that incorporating bile salt edge activators into hexosomes can significantly improve their skin penetration properties [127]. Aquasomes are composed of three layers: A solid nanocrystalline core, an oligomeric shell, and a layer of a bioactive substance absorbed onto the shell [111]. They are produced via colloidal precipitation, plasma condensation, and inverted magnetron sputtering. Aquasomes have high drug loading capacity and can protect fragile drug molecules from degradation [128]. Colloidosomes are typically used to encapsulate sensitive bioactive compounds and are hollow shell microcapsules created of coagulated particles [111]. Sphingosomes are comprised of sphingolipids (sphingosine, ceramide, etc.) and are concentric, bilayered nanovesicles with an acidic pH inside [113]. The resultant vesicular systems can be unilamellar, multilamellar, oligolamellar or multivesicular. Different preparation methods such as reverse phase evaporation, mechanical dispersion, solvent injection, sonication, and microfluidization can be used for the production of sphingosomes. They are characterized by the enhanced drug loading efficiency and stability [129]. Ufasomes are composed of lipid bilayers derived from unsaturated fatty acids and ionic surfactants [130]. In parallel with the conventional liposomes, they are more stable and have a better drug loading efficiency, but are more susceptible to oxidation [131].

### 5.2. Nanovesicular Carriers in the Treatment of Skin Disorders

#### 5.2.1. Antipsoriatic Effect

Psoriasis is a skin disorder characterized by impaired epidermal differentiation, commonly treated by systemic methotrexate, an effective cytotoxic drug. Abdelbary and Abou Ghaly generated topical methotrexate-loaded niosomes for the influence on psoriasis [132]. A thin-film hydration technique is used for the preparation by the inclusion of a surfactant (Span 60) and cholesterol. In comparison with the free drug solution, an increased drug deposition in the skin of rats is monitored.

#### 5.2.2. Antifungal Effect

Perez et al. prepared ultra-deformable liposomes containing amphotericin B to treat cutaneous fungal infections and leishmaniasis [133]. Liposomes containing Tween 80 as an edge activator had maximal deformability and the highest drug/phospholipid ratio. Amphotericin B was encapsulated at 75% encapsulation efficiency in their bilayer. However, drug-loaded liposomes were more toxic to fungal strains than to mammalian cells.

#### 5.2.3. Anti-Vitiligo Effect

Garg et al. developed ethosome-based nanohydrogel formulations of methoxsalen to effectively treat vitiligo with enhanced topical delivery [134]. The formulation contained approximately 28% of ethanol. Ethosomes are incorporated into Carbopol gels and showed substantially skin permeation (on rats), accumulating in epidermal and dermal layers. In addition, there are observed erythema and reduced skin phototoxicity in comparison with a conventional cream.

#### 5.2.4. Anti-Acne Effect

The two main processes that are typical for *acne vulgaris* include:

- The proliferation of *propionibacterium acnes* bacteria in pilosebaceous units of the skin;
- Local inflammation [135].

Traditional topical anti-acne compositions mainly cause burning, erythema, photosensitivity, scaling, and bacterial resistance [136]. In 2008, Touitou et al. developed an ethosomal gel system containing clindamycin phosphate and salicylic acid for an efficient acne treatment and enhanced topical tolerability [135]. Recently, Apriani et al. developed an azelaic acid ethosome-based cream against *propionibacterium acne* [137]. The ethosomal cream demonstrated a superior antibactericidal activity compared with the marketed cream Zelface^®^.

#### 5.2.5. Antiviral Effect

Acyclovir has been investigated for the topical treatment of viral infections for more than four decades [138]. In 1999, Horwitz et al. reported an ethosomal cream system for acyclovir—commercial Zovirax^®^ [139]. Recently, Shukla et al. designed an ethosomal gel with acyclovir, where the ethosomes were prepared by the cold method [140]. The survey shows the in vitro drug release of 82% over 8 h with a zero-order release profile.

#### 5.2.6. Local Anesthetic Effect

In the experimental work, Babaie et al. prepared lidocaine-loaded nanoethosomes for penetration into the deep strata of the skin with a particle size around 100 nm [141]. Increased ethanol concentration from 10 to 40% leads to the production of ethosomes with four-times larger particle sizes.

#### 5.2.7. Antibiotic Effect

In 2005, Godin et al. developed an ethosomal system for the dermal delivery of antibiotics to improve their penetration through the SC and the bacterial membrane/cell wall [142,143]. In addition, Zahid et al. formulated ethosomes containing clindamycin phosphate in a recently announced report using a cold method [144]. The optimized formulation demonstrated an excellent in vitro drug release.

#### 5.2.8. Anticarcinogenic Effect

A report by Cosco et al. represented the formation of transfersomes for the combined delivery of resveratrol and 5-fluorouracil. The co-encapsulation of the drugs synergistically improved their anti-cancer activity on skin cancer cells [145].

Table 5 presents the practical implementation of nanovesicular carriers in dermal drug delivery systems.

## 6. Solid Lipid Nanoparticles and Nanostructured Lipid Carriers

Solid lipid nanoparticles (SLN), as well as nanostructured lipid carriers (NLC), are extensively employed in cutaneous delivery systems. Since their creation in the nineties, lipid nanoparticles (SLN and NLC) are well known by the research and pharma technology community. Easily available raw materials, relatively simple production methods, biocompatibility, and non-toxicity as their advantages over other colloidal carriers can be mentioned as the main reasons for this [67].

Therefore, these two types of lipid nanoparticles—SLN and NLC, are classified according to their structure. First, SLN are developed with a composition of solid lipids only. Then, to upgrade to SLN, NLC were created by representing a mixture of solid and liquid lipids, with a predominant solid lipid [176].

The dermal use of SLN and NLC is proving to be one of the most convenient for therapeutic and cosmetic purposes, despite various applications to date. Lipid nanoparticles are aqueous dispersions with low viscosity for successful direct application to the skin, which implies their incorporation in semisolid systems based on SLN or NLC. One of the first successful administered and marketed products based on NLC is Cutanova^®^ from Dr. Rimpler GmbH (Wedemark, Germany) and Nanobase^®^ from Yamanouchi (Tokyo, Japan) [64].

### 6.1. Solid Lipid Nanoparticles

Generally speaking, SLN are nanometric colloidal carriers composed of a solid lipid core with an incorporated active pharmaceutical ingredient(s) (API) and a surfactant-stabilized shell [177,178,179]. SLN are composed in the 1990s and have unique properties such as biodegradability, impressive toxicity profile, protection of the API against degradation, high load capacity, sterilization ability, and scalability [180,181]. The interaction between the lipid core of SLN and the waxy lipids in SC leads to a significant permeation enhancement of the encapsulated drug into the skin, which determines their successful cutaneous application [182].

### 6.2. Nanostructured Lipid Carriers

The second generation of lipid nanoparticles—NLC, are composed of a mixture of solid lipids and liquid lipids in the nanocore, usually in a ratio of 7:3 to 9:1 [183]. This leads to a more significant disorder of the core of the lipid matrix, and accordingly decreases the melting point to stop the recrystallization of solid lipids [184]. NLC are considered to be an improved variety of SLN, holding the same unique properties, but with an optimized core composition, resulting in a higher drug loading capacity, better stability, and ability to act at lower temperatures. Of note is the fact that NLCs are still solid at body temperature [185].

### 6.3. Preparation of SLN and NLC

The literature describes a significant number of production methods and many different combinations of lipids to obtain SLN and NLC. Nevertheless, the most common technique used today is high-pressure homogenization (HPH). The procedure is divided into two stages:

- Hot homogenization—the lipids are heated above their melting point;
- Cold homogenization—takes place at low temperatures and is suitable for hydrophilic and temperature-sensitive API [186,187].

Other commonly used techniques are: Sonication/ultra-sonication [188,189], membrane contactor technique [190], phase inversion [191], solvent injection [192], emulsification [193], the microemulsion method [194], etc.

### 6.4. SLN and NLC in the Treatment of Skin Disorders

In dermal applications, SLN and NLC create a thin hydrophobic monolayer during skin contact, which has a pointed occlusive effect that settles the API penetration and prevents water loss from the skin [195].

When applied topically, the lipid nanoparticles interact with the sebum and specific skin lipids, provoking a change in the natural arrangement of corneocytes. As a result of this interaction, the encapsulated molecules are released, and their penetration into the lower layers of the epidermis and dermis is potentiated, depending on their lipophilicity, of course [196].

#### 6.4.1. Antioxidant Effect

Okonogi and Riangjanapatee formulated NLC loaded with lycopene through a hot HPH. It has been found that the NLC with the highest concentration of lycopene had the slowest release rate and better antioxidant activity [197].

In another study, Shrotriya et al. reported the development of SLN loaded with resveratrol (entrapment efficiency of 86–89%) to treat irritant contact dermatitis (chronic skin disorder with eczematous injuries). The composition was realized by incorporation into a Carbopol gel and showed increased antioxidant activity compared with a conventional resveratrol gel [198].

Furthermore, Montenegro et al. designed a novel Idebenone (IDE)-loaded NLC containing tocopheryl acetate (VitE) as a liquid component to obtain a synergic effect between IDE and VitE [199].

#### 6.4.2. Anti-Inflammatory Effect

Pivetta et al. formulated NLC with thymol for the local treatment of inflammatory skin diseases (entrapment efficiency of 89%). The NLC were incorporated into a gel and showed anti-inflammatory activity and healing of induced psoriasis in mice [200].

Gad et al. reported the encapsulation of chamomile oil in SLN for the local treatment of wounds. The composition contained stearic acid and chamomile oil and was prepared by the method of hot homogenization. Wound reduction was shown in the topical application in rats [201].

#### 6.4.3. Antifungal Effect

Butani et al. developed a stable SLN system, containing amphotericin B with an enhancing antifungal activity (entrapment efficiency of 94%). The formulation indicated higher drug permeation and drug accumulation in the skin than the conventional gel in rats. A solvent diffusion technique was used for the preparation of the SLN [202].

NLC, for the treatment of candidiasis with Mediterranean essential oils and clotrimazole, were designed by Carbone et al. As a result, they are obtained as a stable NLC, without an initial burst effect and with prolonged release of clotrimazole, as well as an enhanced antifungal activity [203].

#### 6.4.4. Anti-Acne Effect

Tretinoin-loaded NLC with anti-aging and anti-acne activities were reported by Ghate et al. The hot melt probe sonication and hot melt microemulsion methods were used to prepare the NLC. The tretinoin-loaded NLC in Carbopol gels showed no irritation or erythema after the application in rats [204].

Malik and Kaur developed the azelaic acid-loaded NLC, prepared by the melt emulsification and ultra-sonication method (entrapment efficiencies greater than 80%). NLC were incorporated into aloe-vera-based Carbopol hydrogels and demonstrated a deeper skin penetration than the commercial product (Aziderm 10%). Furthermore, the in vivo experiment in mice showed a higher effect of NLC incorporated into a gel than the plain drug suspended in the gel [205].

Table 6 presents the practical implementation of SLN and NLC in dermal drug delivery systems.

## 7. Microemulsions and Nanoemulsions

In general, microemulsions and nanoemulsions are dispersion systems composed of two immiscible liquid phases that can penetrate deeper levels of the skin [226]. Evidence suggests that they may disrupt the SC lipid structural order, resulting in the loss of skin barrier properties [227]. Despite the apparent similarities between these two systems representing low-viscosity colloidal dispersions, they are classified as entirely different formulations [228].

### 7.1. Microemulsions

It has been found that microemulsions are spontaneously formed, transparent, and isotropic thermodynamically stable dispersion systems. The composition of the droplets is carried out by the precise mixing of volumes of immiscible liquids (usually oil and water) and the interfacial film of stabilizing surfactants at specific pressures and temperatures [229]. Short alkyl chain alcohols such as co-surfactants are typically chosen to potent the spontaneous formation of microemulsions. The isotropic and visually mono-phasic transparent system, in which the droplet size is usually below 100 nm, creates a flexible interfacial film characterized by ultra-low surface tension values [230]. The microemulsion as a formulation can improve the delivery of skin MP with both hydrophilic and lipophilic active substances compared with conventional carriers. In this regard, Patel et al. reported that the microemulsion with ketoconazole penetrated more efficiently than the saturated aqueous solution [231].

Three various structural types of microemulsions can be formed:

- Oil-in-water (O/W) microemulsion;
- Water-in-oil (W/O) microemulsion;
- Bicontinuous microemulsion [232].

### 7.2. Nanoemulsions

Nanoemulsions typically contain 20–500 nm large droplets and have a different appearance depending on their size. Traditionally, they are stabilized by surfactants and do not change in the long term [233]. However, they are non-equilibrium structures, and an energetic input has to be typically applied (often from an emulsion) to form the droplet size according to the nanoscale [234]. The basic methods for preparing nanoemulsions are high-energy emulsifying methods such as HPH, ultrasound, jet spraying, microfluidization, and low energy emulsifying methods such as solvent displacement, spontaneous emulsification, phase inversion [235].

### 7.3. Microemulsions and Nanoemulsions in the Treatment of Skin Disorders

#### 7.3.1. Antipsoriatic Effect

The clobetasol propionate- and calcipotriol-loaded nanoemulsion gel for the topical treatment of psoriasis is reported, developed, and optimized by Kaur et al. The spontaneous emulsification method was used for the preparation. Compared with the other commercial MP [236], the nanoemulsion containing gel showed higher antipsoriatic activity in mice.

Recently, Rajitha et al. reported the preparation of loaded nanoemulsion based on chaulmoogra oil, which is based on the self-emulsification method. Compared with the conventional methotrexate solution, the nanoemulsion showed enhanced skin permeation and retention of methotrexate in the deep skin layers [237].

#### 7.3.2. Antifungal Effect

In another study, Coneac et al. reported the development of microemulsion-loaded hydrogels for the topical delivery of fluconazole. Nonionic surfactants have been used to stabilize the microemulsions, which then were incorporated in Carbopol gels. Compared with the conventional hydrogel and Nizoral^®^ cream, the optimized microemulsion-loaded hydrogels showed higher in vitro flux values, higher release rate, and higher in vitro antifungal activity against Candida albicans [238].

#### 7.3.3. Anti-Inflammatory Effect

Two nanoemulsion systems for the dermal application of natural or synthetic mixtures of pentacyclic triterpenes, with an anti-inflammatory effect, were reported by Alvarado et al. [239]. Slightly different permeation profiles of natural and synthetic triterpene-containing formulations are stated. The nanoemulsion, containing a natural triterpene mixture, demonstrated a more significant anti-inflammatory activity due to the slower permeation through the mouse skin [239].

In another study, Goindi et al. reported an ionic liquid-in-water microemulsion formulation that can solubilize etodolac, a poorly water-soluble anti-inflammatory drug. An effective permeation profile, as well as anti-arthritic and anti-inflammatory activities are evaluated in vivo in different models compared with a marketed formulation of etodolac (Proxym gel^®^) [240].

#### 7.3.4. Antioxidant Effect

Lv et al. reported the preparation of essential oil-based microemulsions for topical application in order to improve the solubility, photostability, and skin permeation of quercetin. First, self-micro emulsifying DDS were prepared and then formed microemulsions. The microemulsions protected quercetin from degradation in an alkaline environment and under UV radiation. In these formulations, the in vitro skin permeation study on rats showed 2.5–3 times enhanced permeation capacity of quercetin compared with the conventional aqueous solution [241].

#### 7.3.5. Local Anesthetic Effect

To optimize the percutaneous absorption of lidocaine and prilocaine, Negi et al. formed nanoemulsions using the high-shear mixing method followed by the HPH. The optimized nanoemulsion systems showed higher permeation rates and permeability coefficient values in parallel with the marketed cream and were further incorporated into a Carbopol hydrogel. In addition, the nanoemulsions and the nanoemulsion gel had a stronger anesthetic effect in vivo than the commercial product [242].

#### 7.3.6. Anticarcinogenic Effect

In the last few years, Pham et al. developed a nano-emulsification approach to optimize the incorporation of Tocomin^®^ for the accompanying therapy of skin carcinomas. Different preparation methods were used. The technique, combining a single-phase in-version temperature homogenization method with ultrasonication, produced a stable Tocomin^®^-loaded nanoemulsion, which demonstrated an exceeding cytotoxic profile against two human cutaneous carcinoma cell models [243].

Table 7 presents the practical implementation of microemulsions and nanoemulsions in dermal drug delivery systems.

## 8. Topical Dosage Forms with Lipid Nanoparticulate DDS for the Treatment of Skin Disorders

The size of Global Topical Drug Delivery Market was estimated at USD 95.08 billion in 2020 and expected to reach USD 101.10 billion in 2021 and USD 140.01 billion by 2026. The current market situation (USA, EUR, JP, AUS) for the topical skin products, shows domination of generic products—about 74% of all the approved topical products are generic equivalents of reference medicines (RLD). According to the collected data, gels, creams, ointments, lotions, and solutions dominate the market for both topical reference and topical generic products. The available semi-solid, solid, and liquid topical products contain different combinations of surfactants, oils, water, colloidal, and solid ingredients in solutions or dispersions [263].

Specific needs of skin, affected by inflammation, acne, or infections require adequate drug therapy with appropriate topical dosage forms. For example, the adhesive patch can provide a sustained and controlled release, while the gel can provide a faster and more intense action. On the other hand, some topical dosage forms may not be most suitable for application to certain areas of the skin, around the eyes, for example [264].

One of the most important considerations in the development of the topical dosage forms is the patient need. The second consideration is the drug’s physicochemical properties. In general, regulatory procedures for the registration of topical products are slow, as clinical equivalence studies (clinical trials) involve a high number of participants, require time and significant costs to ensure a sufficiently objective assessment of the final therapeutic effect. Technological or cost problems are among the reasons for additional difficulties in the implementation of promising dermatological products [265].

Table 8 summarizes some of the literature available on topical nano-based topical dosage forms for skin diseases.

## 9. Future Prospects of Lipid Nanoparticulate DDS for the Treatment of Skin Disorders

It can be generalized that the main challenges in the development of cutaneous lipid nanometric delivery systems include:

- Precise delivery across the skin and to certain skin strata, depending on the final target;
- Successful elimination of lipid nanomaterial toxicity threats in topical medical formulations and cosmetics;
- Ensuring improved permeation and low skin irritability as a result of the use of lipid nanocarriers;
- Improved cutaneous release of incorporated API with a broad spectrum of physiological and physicochemical properties.

Lipid nanoparticulate DDS can be employed intensively for the delivery of phytomedicines intended for topical administration. The approach can be promising in this regard, considering the difficulties in their delivery which is caused by their physicochemical properties.

The formulation of phytopreparations with lipid nanoparticles would find a useful application in nanomedicine at the desired targeted delivery, for example, in cancer treatments.

## 10. Conclusions

Skin disorders represent a progressively emerging clinical public health problem. Treatment strategies based on conventional formulations are non-specific and can lead to considerable systemic toxicity. The progressive approach of the use of lipid nano-formulations as skin drug delivery systems can provide an incomparable prospect for the application of highly competent and safe treatments with the improved benefit-risk ratio.

The use of lipid nanoparticlulate DDS is favored recently due to the GRAS status of the excipients. Lipid nanocarriers can effectively protect the API from degradation on the skin’s surface, increase their concentration gradient in the upper skin layers, and enable gradual release. Lipid nanoparticles for topical application could be formulated with the high content of lipid matrix or dispersed in different foundations.

Lipid nanosystems provide a promising, flexible platform for the safe, effective, and biocompatible topical delivery of the API, as they do not cause cytotoxicity or morphological changes in the skin layers. The interest shown by pharmaceutical scientists, in the development of lipid nanoparticle delivery systems, may offer a future that provides sufficiently efficient lipid nanoparticle products for needy users.

## Figures and Tables

**Table 1:** Mostly used therapeutic solutions for T cell-mediated skin disorders.

Drug Substance	Main Action	References
Corticosteroids	For local application. Manifests a slight immunosuppressive effect. Ineffectiveness in severe cases.	[49]
Retinoids	Retinoids can be synthetic or natural derivatives of vitamin A. Probable mechanisms of action: Facilitate the transport of cytoplasmic retinoid-binding proteins; influence angiogenesis; modulate T cell responses.	[50,51]
Vitamin D3 metabolites	Metabolites of vitamin D3 are included in ointments and creams for the treatment of psoriasis. Good effects in milder diseases. Not fully understood effects. The 1,25(OH)2D3 enhances the suppressive activity of CD4(+)CD25(+) cells in draining lymph nodes.	[52,53,54]
UVB treatment	Multiple effects and effectiveness for several T cell-mediated skin diseases. Not equally effective in the different disorders. Unspecific and generally immunosuppressive.	[55,56,57]
Methotrexate	Immunosuppressive effect. It does not target specific T cell groups. Still not a fully understood therapeutic effect.	[49,51]
Cyclosporine A	Affects IL-2 producing cells, in particular CD4(+) T cells. General immunosuppressive effect.	[49,51]

**Table 2:** Presentation of some lipid-based delivery systems.

Lipid-Based Delivery System	Description	Advantages	Disadvantages
Nanovesicular carriers			
Liposomes [68]	Conventional single or multilayer vesicles. Formed by contact of biodegradable lipids with an aqueous medium. Widely used as drug carriers for hydrophilic and lipophilic molecules.	Biocompatible and biodegradable lipids. Conventional production processes. Improved local delivery. Suitable for loading both hydrophobic and hydrophilic substances.	Insufficient chemical and physical stability. Short half-life. Inadequate penetration into the viable epidermis and dermis. High production costs. Difficulties in scalability.
Transfersomes [69,70,71]	Highly deformable, elastic or ultra-flexible liposomes. Vesicles, similar to conventional liposomes in terms of preparation and structure. Claimed to permeate as intact vesicles through the skin layers. Functionally deformed due to the presence of an edge activator.	Smaller vesicle size, higher elasticity. Compared with conventional liposomes—better penetration through the skin. High membrane hydrophilicity and elasticity allow them to avoid aggregation and fusion under osmotic stress, unlike the conventional liposomes.	Elasticity of these vesicles can be compromised by hydrophobic drug loading. Occlusive application and complete skin hydration limit transdermal delivery due to inhibition of transdermal hydration. Relatively high production costs. Absence of well-established regulatory guidance for skin delivery.
Ethosomes [72,73]	Lipid vesicles are composed of phospholipids, ethanol, and water. Similar to liposomes in terms of their preparation techniques and structure. Concentration of ethanol 20–45%. Their size decreases with an increase in the ethanol concentration. Exhibit high encapsulation efficiency.	Appropriate for both hydrophobic and hydrophilic drug loading. Enhanced skin delivery under both occlusive and nonocclusive conditions. Higher elasticity, smaller vesicle size, and higher entrapment efficiency than conventional liposomes.	High ethanol content can lead to skin irritation and toxicity. Possible structural and chemical instability during long-term storage. Need to optimize the concentration of lipids and ethanol for improved physicochemical properties and stability of ethosomes.
Lipid nanoparticulate carriers			
Solid lipid nanoparticles [74,75]	Colloidal lipid nanoparticles are composed of a physiological biodegradable solid lipophilic matrix (solid at room temperature and body temperature), in which the drug molecules can be incorporated.	Increased drug stability. High drug payload. Incorporation of lipophilic and hydrophilic drugs. Avoidance of organic solvents. Lack of biotoxicity of the carrier. Relatively cost-effective.	SLN are incorporated into semisolid carriers such as ointments and gels due to the high water content. Potential expulsion of active compounds during storage. Cost-effective manufacturing process.
Nanostructure Lipid Carriers [76,77]	Colloidal lipid nanoparticles composed of physiological mixing liquid lipid (oils) with the solid lipids, in which the liquid lipid is incorporated into the solid matrix or localized at the surface of solid particles	Improved drug loading compared with SLN. Lower water content compared with SLN. Firmly incorporates the drug substance during storage. Biodegradable and biocompatible. Large-scale production is easily possible.	Tendency to unpredictable gelation. Polymorphic transition. Low drug incorporation due to the crystalline structure of solid lipids. Lack of long-term stability data.
Lipospheres [78,79,80]	Microspheres, composed of solid hydrophobic lipid core and stabilized by a monolayer of a phospholipid embedded on the surface.	Improved drug stability, especially for photo-labile drugs. Possibility for controlled drug release. Controlled particle size. High drug loading. Biodegradable and biocompatible.	Larger particle size and poor skin permeation compared with lipid-based vesicular carriers, SLN, and NLC. Poor drug loading for hydrophilic compounds.

**Table 3:** Presentation of some of the patents for the application of ethosomes in skin formulations.

Application	Title/Inventors	Year	Results
CN103006562 (A)	Daptomycin ethosome preparation/ Li Chong, Liu Xia, Yin Qikun, Wang Xiaoying, Chen Zhangbao	2013	Stable translucent dispersion system with a small and uniform particle size. High entrapment efficiency. Excellent transdermal performance. Simple and convenient preparation method.
EP 2810642 A1	Chitosan-modified ethosome structure/ Chin-Tung Lee, Po-Liang Chen	2013	The chitosan-modified ethosome structure contains different active substances. Improved storage and transportation.
CN103800277 (A)	Leflunomide ethosome composition and its preparation method/ Zhang Tao, Ding Yanji, Deng Jie, Luo Jing, Zhong Xiaodong	2014	Improves the transdermal rate of leflunomide. Improves curative effects.
CN103536700 A	Chinese medicinal ethosome gel patch for treating herpes zoster and preparation method thereof/Bu Ping, Hu Rong, Chen Lin, Wei Rong, Wu Huanhuan, Huang Xiaoli	2014	Easy in medication. Convenient to use. Good therapeutic effect. Strong analgesic action. No adverse reaction.
CN 104706571 A	Preparation method of ethosome/ natural material/polyvinyl alcohol composite hydrogel/Yang Xingxing, Lynn, Chen Mengxia, Fanlin Peng	2015	Addition of the polyvinyl alcohol, which improves the properties of the hydrogel.
CN106474065A	A kind of tetracaine ethosome and its preparation technology/Zhu Xiaoliang, Wu Dongze, Ma Xiaodong	2017	Stable in terms of component and proportion. Preferable percutaneous permeation.

**Table 4:** Emerging lipid nanovesicular carriers for skin drug delivery.

Emerging Lipid Vesicles	Description	Reference
Niosomes	Nonionic surfactant and cholesterol (or its derivatives)—based vesicle with improved stability (especially oxidative stability).	[105,106]
Cubosomes	Submicron, nanostructured particles, composed of bicontinuous cubic liquid crystalline phase.	[107,108,109]
Hexosomes	Constructed of hexagonal liquid crystalline phases dispersed in a continuous aqueous medium.	[110]
Aquasomes	Self-assembled nanovesicles, composed of three layers.	[111]
Colloidosomes	Hollow shell microcapsules composed of coagulated particles.	[112]
Sphingosomes	Contained sphingolipids such as sphingosine, ceramide, sphingomyelin or glycosphingolipid; and are concentric, bilayered nanovesicles with an acidic pH inside.	[113]
Ufasomes	Lipid carriers attach to the surface of the skin and support the lipid exchange between the outermost layers of the SC.	[114,115]
Archeosomes	Consisted of archebacteria lipids, chemically distinct from eukaryotic and prokaryotic species. Less sensitive to high temperature, alkaline pH, and oxidative stress.	[116,117]
Lipoplexes	Cationic lipid-DNA complexes. Efficient carriers for cell transfection. Toxic effects arising from either cationic lipids or nucleic acids.	[118]
Proliposomes	Dry, free-flowing particles that immediately form a liposomal dispersion in contact with water.	[119,120]

**Table 5:** Application of nanovesicular carriers in dermal DDS for the treatment of skin disorders.

LNP Type	API/Drug	Application	Reference
Conventional liposomes	Licorice	Licorice-loaded liposomes included in the formulation for the treatment of oxidative stress injuries.	[146]
Conventional liposomes	Quercetin and resveratrol	Quercetin- and resveratrol-loaded liposomes for the treatment of inflammatory/oxidative responses associated with skin cancer.	[147]
Liposomes	Tretinoin	A tretinoin-loaded liposomal formulation for the treatment of acne.	[148]
Liposomes	Benzoyl peroxide	Benzoyl peroxide and chloramphenicol encapsulation in liposomes for the treatment of acne.	[149]
Liposomes	Benzoyl peroxide/Adapalene	Benzoyl peroxide- and adapalene-loaded modified liposomal gel for the treatment of acne.	[150]
Transfersomes	Indocyanine green	Indocyanine green-loaded transfersomes for the treatment of acne vulgaris.	[151]
Transfersomes	5-Fluorouracil	5-Fluorouracil-loaded transfersomes for the treatment of skin cancer.	[152]
Transfersomes	Resveratrol and 5-fluorouracil	Transfersomes containing resveratrol and 5-fluorouracil for the treatment of skin cancer.	[145]
Transfersomes	Amphotericin B	Development of amphotericin B-loaded transfersomes for antifungal and antileishmanial treatment.	[133]
Transfersomes	siRNA	Transfersomes containing siRNA developed for delivery to the human basal epidermis for the treatment of melanoma.	[153]
Transfersomes	RNAi	Transfersomes containing RNAi, formulated for the treatment of psoriasis.	[154]
Transfersomes	Indocyanine	Indocyanine-loaded transfersomes for the treatment of basal cell carcinoma.	[155]
Transfersomes	Clindamycin	Development of clindamycin-loaded transfersomes for the treatment of acne.	[156]
Transfersomes	Paclitaxel	Paclitaxel containing transfersomes, modified by a cell-penetrating-peptide embedded in oligopeptide hydrogel for the topical treatment of melanoma.	[157]
Transfersomes	Sodium stibogluconate	Transfersomes loaded with sodium stibogluconate for the treatment of leishmaniasis.	[158]
Transfersomes	Lidocaine	Lidocaine transferosomal gel, containing permeation enhancers for local anesthetic action.	[159]
Transfersomes	Sulforaphane	Transfersomes comprising sulforaphane for the treatment of skin cancer.	[160]
Transfersomes	Miltefosine polyphenol	Formulation of miltefosine polyphenol-loaded transfersomes for the topical treatment of leishmaniasis.	[161]
Transfersome	N-acetylcysteine	N-acetylcysteine-loaded transfersomes for antioxidant activity in anti-aging therapy.	[162]
Ethosomes	Methoxsalen	Formulation of ethosomes containing methoxsalen for the topical treatment against vitiligo.	[134]
Ethosomes	Griseofulvin	Design of griseofulvin-loaded ethosomes for enhanced antifungal treatment.	[163]
Ethosomes	Cryptotanshinone	Cryptotanshinone-loaded ethosomes for anti-acne treatment.	[164]
Ethosomes	Epigallocatechin-3-gallate	Epigallocatechin-3-gallate-loaded ethosomes for the treatment of skin cancer.	[165]
Ethosomes	Thymoquinone	Thymoquinone-loaded ethosomes for the topical treatment of acne.	[166]
Ethosomes	Clobetasol propionate	Ethosomes of clobetasol propionate for the treatment of eczema.	[167]
Ethosomes	Tretinoin	Gel containing tretinoin-loaded ethosomes for anti-acne treatment.	[168]
Ethosomes	Azelaic acid	Azelaic acid-loaded ethosomes for anti-acne treatment.	[137]
Niosomes	Resveratrol	Resveratrol-loaded niosomes for the treatment of psoriasis.	[169]
Niosomes	Diacerein	Niosomes for the topical diacerein delivery and treatment of psoriasis.	[170]
Niosomes	Celastrol	Celastrol-loaded niosomes for the treatment of psoriasis.	[171]
Cubosomes	Paclitaxel	Paclitaxel-loaded cubosomes against skin cancer.	[172]
Cubosomes	Erythromycin	Erythromycin-loaded cubosomes for the treatment of acne.	[173]
Hexosomes, cubosomes	Ketoconazole	Ketoconazole-loaded hexosomes for antifungal treatment.	[174]
Ufasomes	Minoxidil	Minoxidil-loaded ufasomes for the treatment of hair loss.	[175]

**Table 6:** Application of SLN and NLC DDS for the treatment of skin disorders.

LNP Type	API/Drug	Application	Reference
SLN	Doxorubicin	Doxorubicin-loaded SLN for the treatment of skin cancer.	[206]
SLN	Adapalene	Adapalene-loaded SLN in the gel for anti-acne treatment.	[207]
SLN	Triamcinolone acetonide	Triamcinolone acetonide-loaded SLN for the topical treatment of psoriasis.	[208]
SLN	Resveratrol, vitamin E, and epigallocatechin gallate	SLN containing resveratrol, vitamin E, and epigallocatechin gallate for antioxidant benefits.	[209]
SLN	Silybin	Silybin-loaded SLN enriched gel for irritant contact dermatitis.	[210]
SLN	Fluconazole	Fluconazole-loaded SLN topical gel for the treatment of pityriasis versicolor.	[211]
SLN	Tazarotene	Tazarotene-loaded SLN for the treatment of psoriasis.	[212]
SLN	Miconazole nitrate	Miconazole nitrate-loaded SLN for antifungal activity.	[213]
SLN	Adapalene	Adapalene-loaded SLN for anti-acne therapy.	[214]
SLN	Isotretinoin and α-tocopherol	SLN loaded with retinoic acid and lauric acid for the topical treatment of acne vulgaris.	[215]
NLC	Spironolactone	Spironolactone-loaded NLC-based gel for the effective treatment of acne vulgaris.	[216]
NLC	Clobetasol propionate	NLC-based topical gel of clobetasol propionate for the treatment of eczema.	[217]
NLC	Tacrolimus and tumor necrosis factor α siRNA	NLC co-delivering tacrolimus and tumor necrosis factor α siRNA for the treatment of psoriasis.	[218]
NLC	Itraconazole	Topical NLC containing itraconazole for the treatment of fungal infections.	[219]
NLC	Apremilast	NLC for topical delivery of apremilast for the treatment of psoriasis.	[220]
NLC	Dithranol	Dithranol-loaded NLC-based gel for the treatment of psoriasis.	[221]
NLC	Voriconazole	Voriconazole-loaded NLC for antifungal applications.	[222]
NLC	Mometasone furoate	NLC-based hydrogel of mometasone furoate for the treatment of psoriasis.	[223]
NLC	Antimicrobial peptide nisin Z	Antimicrobial peptide nisin Z with conventional antibiotic-loaded NLC to enhance antimicrobial activity.	[224]
NLC	Adapalene and vitamin C	Adapalene- and vitamin C-loaded NLC for acne treatment.	[225]

**Table 7:** Application of microemulsions and nanoemulsions DDS for the treatment of skin disorders.

Type	API/Drug	Application	Reference
Microemulsion	Tazarotene	Tazarotene-loaded microemulsion for the treatment of psoriasis.	[244]
Microemulsion	Methotrexate	Methotrexate-loaded microemulsion for the treatment of psoriasis.	[245]
Microemulsion	Retinoid	Retinoid-loaded microemulsion for the treatment of psoriasis.	[246]
Microemulsion	Clotrimazole	Microemulsion coated with chitosan and containing clotrimazole for antifungal activity.	[247]
Microemulsion	Griseofulvin	Griseofulvin-loaded microemulsion for the antifungal treatment.	[248]
Microemulsion	Boswellia carterii oleo-gum-resin	Boswellia carterii oleo-gum resin-loaded microemulsion for the treatment of acne and eczema.	[249]
Microemulsion	Indian pennywort, walnut, and turmeric	Topical dosage microemulsion of Indian pennywort, walnut, and turmeric for the treatment of eczema.	[250]
Microemulsion	Triamcinolone	Microemulsion containing triamcinolone for transdermal delivery for the treatment of eczema.	[251]
Microemulsion	Retinyl palmitate	Microemulsion containing retinyl palmitate for the treatment of acne, aging, and psoriasis.	[252]
Nanoemulsion	Triptolide	Triptolide nanoemulsion gels for the treatment of eczema.	[253]
Nanoemulsion	Ivermectin	Nanoemulsion containing ivermectin for the treatment of different types of parasite infestations.	[254]
Nanoemulsion	Cyclosporine	Cyclosporine-loaded nanoemulsion for the treatment of psoriasis.	[255]
Nanoemulsion	Coumestrol /Hydroxyethylcellulose	Nanoemulsion containing coumestrol and hydroxyethylcellulose for the treatment of antiherpes.	[256]
Nanoemulsion	8-Methoxypsoralen	8-Methoxypsoralenloaded nanoemulsion for the treatment of vitiligo and psoriasis.	[257]
Nanoemulsion	Coenzyme Q10	Coenzyme Q10-loaded nanoemulsion as an antioxidant agent.	[258]
Nanoemulsion	Psoralen	Psoralen-loaded nanoemulsion for the treatment of psoriasis and vitiligo.	[259]
Nanoemulsion	Isotretinoin	Isotretinoin-loaded nanoemulsion for the treatment of acne.	[260]
Nanoemulsion	Amphotericin B	Amphotericin B-loaded nanoemulsion for the antifungal treatment.	[261]
Nanoemulsion	Zinc phthalocyanine	Zinc phthalocyanine-loaded nanoemulsion for use in photodynamic therapy for leishmaniasis.	[262]

**Table 8:** Topical nanoformulations used in the treatment of various skin conditions.

Type	API/Drug	Disease	Reference
Nanoemulsion gel	Clobetasol propionate	Treatments of psoriasis.	[266]
Nanoethogel	Amphotericin B	Dermatophytes and fungal infections.	[236]
Nanoemulsion gel	5-Fluorouracil	Non-melanoma skin cancers.	[267]
Hydrogel	Zinc oxide	Wound healing effect on fibroblast cells.	[268]
Nanoemulgel, NLC	Vitamin E	Skin hydration.	[269]
Hydrogel	Cyclosporine and calcipotriol	Treatments of psoriasis.	[270]

## Data Availability

Data is contained within the article.
